# Assessing gut microbial provisioning of essential amino acids to host in a murine model with reconstituted gut microbiomes

**DOI:** 10.21203/rs.3.rs-6255159/v1

**Published:** 2025-03-24

**Authors:** Paul Ayayee, Gordon Custer, Jonathan B. Clayton, Jeff Price, Amanda Ramer-Tait, Thomas Larsen

**Affiliations:** 1 Department of Biology, University of Nebraska at Omaha, Omaha, NE, USA.; 2 Department of Natural Sciences, University of Maryland Eastern Shore, Princess Anne, MD, USA; 3 Department of Food Science and Technology, University of Nebraska-Lincoln, Lincoln, NE, USA; 4 Nebraska Food for Health Center, University of Nebraska-Lincoln, Lincoln, NE, USA; 5 Max Planck Institute of Geoanthropology, Jena, Germany; 6 Institute for Prehistoric and Protohistoric Archaeology, University of Kiel, Kiel, Germany

**Keywords:** Gut microbiome, mouse, auxotrophy, EAA provisioning, stable isotope, germ-free

## Abstract

Gut microbial essential amino acid (EAA) provisioning to mammalian hosts remains a critical yet poorly understood aspect of host-microbe nutritional interactions, with significant implications for human and animal health. To investigate microbial EAA contributions in mice with reconstituted gut microbiomes, we analyzed stable carbon isotopes (^13^C) of six EAAs across multiple organs. Germ-free (GF) mice fed a high-protein diet (18%) were compared to conventionalized (CVZ) mice fed a low-protein diet (10%) following fecal microbiota transplantation 30 days prior and a 20-day dietary intervention. We found no evidence for microbial EAA contributions to host tissues, with ^13^C-EAA fingerprinting revealing nearly identical patterns between GF and CVZ organs. Both groups maintained their expected microbiome statuses, with CVZ gut microbiota dominated by Firmicutes and Bacteroidetes phyla. These findings raise important questions about the functional capacities of reconstituted gut microbiomes. Future studies should investigate longer adaptation periods, varied dietary protein levels, and complementary analytical techniques to better understand the context-dependent nature of microbial EAA provisioning in mammalian hosts.

## Introduction

The gut microbiome produces a range of metabolites that influence host health and physiology, including short-chain fatty acids (SCFAs), neurotransmitters, and essential amino acids (EAAs)^1–3^. SCFAs, such as butyrate, acetate, and propionate, are well-documented for their roles in host energy metabolism, immune modulation, and brain function through their ability to cross the blood-brain barrier^4,5^. In contrast, EAAs - critical for protein synthesis and numerous cellular functions - must be obtained from dietary sources or potentially synthesized de novo by gut microbes for host assimilation. However, the extent to which microbial EAAs contribute to host amino acid pools remains unclear and is likely context-dependent^6,76^. Unlike SCFAs, which are readily absorbed through specific transporters in the colonocytes, microbial EAAs have complex biosynthetic pathways and are produced intracellularly^8^, and are released from gut bacteria following lysis and absorbed in the host’s gastrointestinal tract, particularly the jejunum in the small intestine^9^. This process is constrained by physiological factors such as limited amino acid absorption in the large intestine, which harbors significantly more gut bacteria by biomass than the small intestines^10^, requiring further studies about the functional relevance of microbial EAA provisioning under typical dietary conditions.

Studies investigating gut microbial EAA provisioning rely on various isotope-based approaches. Early methods involved adding isotope labels such as ^14^C or ^15^N to diets or specific metabolites^6,11^. A more recent approach has been to compare the natural abundance concentrations of ^13^C (i.e. without isotope additions/enrichments) between the EAAs in consumer tissue and dietary protein sources^12–14^. Assuming dietary equilibrium, an enrichment in δ^13^C of EAAs in sampled animal tissues relative to those of the diet would indicate gut microbial EAA provisioning. This is attributed to the release of ^13^C-depleted CO_2_ during catabolism, resulting in ^13^C-enrichment of the remaining carbon pool. This process creates a detectable isotopic signal, even when dietary sources are isotopically similar. A significant Δδ^13^C offset (δ^13^C_consumer EAA_ - δ^13^C_diet EAA_) beyond an analytical margin, typically exceeding 1‰, would typically indicate gut microbial EAA sources^13,14^. The degree of ^13^C enrichment would also depend on the δ^13^C values of macronutrients serving as metabolic precursors for EAA synthesis.

In the last decade, a method termed δ^13^C_-EAA_ fingerprinting has been increasingly implemented to infer the biosynthetic origins of de novo synthesized EAAs and to quantify gut microbial contributions^14–23^. A distinct advantage of the δ^13^C_-EAA_ fingerprinting approach is that it can be used in situ to quantify gut microbial contributions to host^15,24^. Recent research based on δ^13^C fingerprinting of EAAs in mice estimated high gut microbial contributions, approximately 60% for valine and 40% for isoleucine when on diets with low protein content (9%)^25^. These estimated contributions substantially exceed those reported in a feeding study with pigs by Torrallardona et al. (2003)^11^ that used a different approach with ^15^N and ^14^C labels, where only 10% of the pigs’ lysine requirements were provided by gut microbes. However, these previous mammalian studies did not utilize germ-free controls, limiting their capacity to attribute observed isotopic offsets to microbial sources definitively.

**Figure 1.**
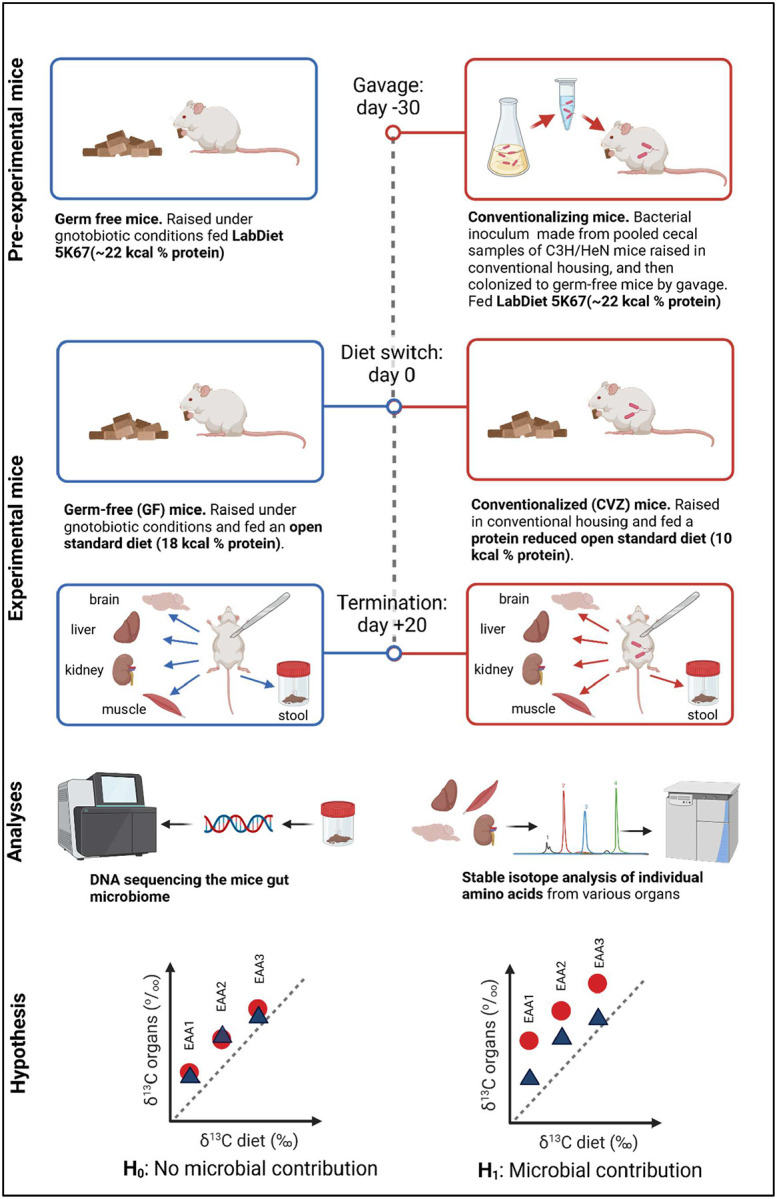
Conceptual diagram illustrating the experimental design and analysis workflow used in this study. Germ-free (GF) mice were raised under gnotobiotic conditions and fed an open standard diet (18 kcal% protein), while conventionalized (CVZ) mice were raised in conventional housing and fed a protein-reduced open standard diet (10 kcal% protein). Both groups were terminated 20 days after the diet switch, and brain, liver, kidney, muscle, were dissected for stable isotope analysis of individual amino acids and stool samples were taken for DNA sequencing of the gut microbiome. Created in BioRender. Larsen, T. (2025). https://BioRender.com/l14k831

Our study addresses this limitation by comparing germ-free mice with conventionalized mice that received fecal microbiota transplants (FMT)^26,27^, directly assessing microbial EAA contributions to the host (see Fig. 1 for a schematic overview of the experimental design). This approach provides insight into the functional capacities of experimentally reconstituted gut microbiomes regarding EAA provisioning to the host^28–30^. Reconstituted microbiomes are increasingly employed as experimental models to elucidate host-microbe interactions and inform microbiome-based therapeutic strategies. Yet, whether these microbiomes fully recapitulate the functional capabilities of naturally developed communities remains unclear. Differences in colonization history, microbial community assembly dynamics, or functional lag times could result in incomplete or altered microbial functionality compared to naturally colonized hosts^29,31,32^. Thus, explicitly assessing the putative nutritional functions of reconstituted microbiomes is essential for translating laboratory findings into broader biological and clinical contexts.

In this study, we compared gut microbial EAA contributions in germ-free (GF) mice lacking a gut microbiome and conventionalized (CVZ) mice with reconstituted FMT microbiome, which ostensibly function normally. Both groups were fed the same diet with one protein source, lactic acid casein, but the CVZ mice received a diet with overall lower protein content than the GF mice (10 vs. 18 kcal% protein, respectively). Given previous evidence of gut microbial EAA provisioning under protein-restricted but not deficient diets^11,25^, we hypothesized that CVZ mice would exhibit discernible differences in δ^13^C-EAA values relative to GF mice and have δ^13^C-EAA patterns indicative of microbial contributions to host.

## Methods

### Experimental design

We used natural variations in stable carbon isotope ratios of essential amino acids (EAAs) δ^13^C_-EAA_ to examine whether gut microbiota supply EAAs to mice (Fig. 1). Germ-free (GF) C57BL/6 (B6) (n= 10) mice were born and reared in flexible film isolators and maintained under gnotobiotic conditions by the Nebraska Gnotobiotic Mouse Program (NGMP) using established protocols^33^. Half of the GF mice (n = 5) were transferred to individually ventilated cages and conventionalized (CVZ) via oral gavage with 100 μL of a conventional in-facility mouse microbiota diluted at 1:10 (w/v; grams of ceca per milliliters of PBS plus 10% glycerol)^33^. The gavage took place 30 days prior to the dietary intervention (see below). The cages featured flooring made of wire mesh, which allowed waste to drop below and out of reach of the animals. The conventional mouse microbiome was obtained initially from pooled cecal samples of C3H/HeN mice raised in conventional housing at the University of Nebraska-Lincoln^33,34^. The remaining GF mice (n = 5) were maintained in flexible film isolators. Before the experiment, all mice were fed an autoclaved diet (LabDiet 5K67) containing ~22 kcal% protein derived from various sources including C4 plant and marine derived proteins (Table S1). At the start of the experiment, both the GF and CVZ mice switched to an irradiated OpenStandard Diet (Research Diets, Inc.) with lactic acid casein (SureProtein^™^ LACTIC CASEIN 720) from grass-fed New Zealand cows as the protein source (Table 1). The mice were reared in The GF mice were fed an OpenStandard diet with 18 kcal% protein (D11112201), and the CVZ mice were fed the same OpenStandard diet but with reduced protein (10 kcal% protein, D22010704–1.5V). The protein level used for the CVZ mice is at the lower end of the protein range (9 – 40 kcal% protein) used by Newsome et al. (2020)^25^ in their study.

We selected a 20-day feeding period for both groups, informed by published data on carbon turnover rates in mice^33,35,36^. We chose a multi-organ approach analyzing the liver, kidneys, muscle, and brain, enabling the detection of potential differences in tissue-specific microbial EAA provisioning or EAA provisioning that might be dependent on metabolic activity. Published data on mouse carbon turnover in single amino acids have shown a replacement of 75%, 60%, 45%, and 30% in the liver, kidneys, muscle, and brain, respectively, within a 20-day period^37^. Twenty days after the diet switch, mice were euthanized, and organs (liver, muscle, kidney, and brain) were collected. Samples were bio-banked at −80°C until further analyses. Putative gut microbial EAA provisioning to the host was investigated by comparing δ^13^C_-EAA_ values between GVZ and GF mice. Significantly higher δ^13^C-EAA in CVZ than CVZ organs would indicate that mice had acquired EAA synthesized by their microbiome (non-dietary sources). Hence, our null hypothesis (H_0_) posits that the gut microbiota does not contribute EAAs to the host, evidenced by an overlap in δ^13^C_-EAA_ values between GF and CVZ mice. The alternative hypothesis (H_1_) posits that the gut microbiota does contribute to EAAs, resulting in higher δ^13^CEAA values in CVZ than in GF mice. We subsequently used the δ^13^C_-EAA_ fingerprinting method to aid in identifying putative gut microbial EAA origins with the expectation that the dietary protein source, lactic acid casein, would classify between bacteria and plants^14^. The Institutional Animal Care and Use Committee at the University of Nebraska-Lincoln approved all procedures involving animals (protocol # 2126).

### Amino acid δ^13^C measurements

Before determining δ^13^C-EAA values, samples were lyophilized under vacuum at −80 °C for 48 hrs. Samples were then pulverized and submitted for analysis at the Stable Isotope Facility at the University of California, Davis (Davis, CA, USA). Briefly, samples were first acid-hydrolyzed for 70 min under a N_2_-gas headspace in 6M HCl at 150°C. Samples were then derivatized as N-acetyl methyl esters via methoxy carbonylation-esterification (NACME)^38,39^. Essentially, derivatized samples were injected into a splitless liner at 260 °C and separated on an Agilent DB-35 column (60 m × 0.32 mm ID × 1.5 μm film thickness) at a flow rate of 2 mL/min under the following temperature program: 70 °C (hold 2 min); 140 °C (15 °C/min, hold 4 min); 240 °C (12 °C/min, hold 5 min); and 255 °C (8 °C/min, hold 35 min). Compound-specific isotope ^13^C-amino acid analysis (δ^13^C_-EAA_) was carried out using a Thermo Trace GC 1310 (GC; Thermo Fisher Scientific, Waltham, MA, USA) coupled to a Delta V Advantage isotope ratio mass spectrometer via the GC IsoLink II (Thermo Electron, Bremen, Germany) (see^39^ for analytical details). All samples were analyzed in duplicate. Replicates of the quality control and assurance reference materials are measured every five samples.

Standard exogenous carbon addition, kinetic isotope effects from derivatization reagents procedures, and normalization to the international reference for δ^13^C VPDB, as well as a calibrated amino acid mixture, UCD AA3, and multiple natural materials, were carried out as per facility practices. δ^13^C-EAA data were obtained for isoleucine, leucine, lysine, phenylalanine, threonine, and valine. The mean standard deviation for all samples was ± 0.2‰, well below the established quality control value of ± 0.4‰. Final accuracy, as determined by the mean absolute difference in the measured and known δ^13^C-EAA values of EAAs from a quality assessment mixture of amino acids, was within ± 0.5‰. Analyses of δ^13^C-EAA values and δ^13^C enrichment among mouse samples and dietary groups were conducted using ANOVA with insect treatment groups and amino acids (all six EAAs) as factors, following normalization to respective dietary δ^13^C_-EAA_ in JMP (SAS). The δ^13^C-offset between mice consumers and their diets^14,40^ can be expressed as follows: Δδ13C = δ^13^C_Consumer EAA_ − δ^13^CDietary EAA.

To identify the biosynthetic origins of EAAs in casein and consumer tissues, we employed linear discriminant function analysis (LDA) based on δ^13^C-EAA training data comprising bacteria, fungi, and plants from Larsen et al. (2013)^41^ after performing interlaboratory calibrations to ensure analytical comparability. With this fingerprinting framework, we predicted the linear discriminant scores of mice tissue and dietary samples. The LDA was executed using the R package MASS (R version 4.2.2.; http://www.R-project.org). We calculated pairwise Bhattacharyya coefficients (BC)^42^ on the LDA-transformed data to quantify the degree of group separation. BCs are a general similarity measure between two multivariate distributions, with 0 indicating no overlap and 1 indicating identical distributions.

### DNA extraction, microbiome sequence generation, and sequence analyses

To verify that CVZ mice had a normal reconstituted gut microbiome and whether GF mice were germ-free, fecal samples from individual mice were used for DNA extraction after the experimental period. Briefly, ~0.25g of all 11 DNA samples were extracted using the DNeasy Blood & Tissue Kit (Qiagen, Germantown, MD, USA) with modifications to the manufacturer’s directions. Samples were submitted for high-throughput paired-end Illumina MiSeq library preparation and sequencing at the University of Nebraska Medical Center Genomics Core. Briefly, a limited cycle PCR reaction was performed on each sample to create a single amplicon, including the V4 (515-F) and V5 (907-R) variable region^43^. The resulting libraries were validated using the Agilent BioAnalyzer 2100 DNA 1000 chip (Agilent), and DNA was quantified using Qubit 3.0 (Qubit^™^, Thermofisher). A pool of libraries was loaded into the Illumina MiSeq at 10 pM. The pool was spiked with 25% PhiX (a bacteriophage) at 10 pM for MiSeq run quality as an internal control [31] to generate 300 bp paired ends with the 600-cycle kit (version 3).

Before processing, primers were removed using Cutadapt (v. 3.7), and acquired reads were processed with the DADA2 package version 1.21^44,45^. Chimeras were removed using the remove chimeraDenovo function, with method = “consensus.” The final sequence table was then trimmed to include only sequences with read lengths ranging from 367 to 375 base pairs. The Silva V138 database was used to assign taxonomy^46^, and the final ASV table, sample metadata, and taxonomic assignments were imported to Phyloseq^47^ for downstream processing. For statistical analyses, all non-bacterial reads and those assigned to chloroplasts or mitochondria were removed before downstream analyses. Raw sequence reads were deposited into the NCBI SRA under BioProject PRJNA927293. All code used for processing raw amplicon reads statistical analyses are at https://github.com/gcuster1991/ayayee_mouse_2023.git. Diversity analyses were performed only for the CVZ mice group, following confirmation of limited to no microbial presence/diversity in the GF mice.

## Results

### Gut microbial EAA supplementation

**Figure 2.**
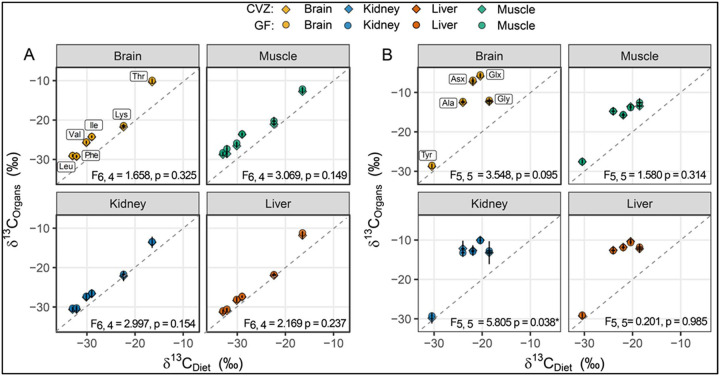
Scatter plot illustrating the relationship between the amino acid δ^13^C values of mouse organs and diets, 20 days after switching from LabDiet 5K67 to an Open Standard Diet. The Open Standard Diet contained 10 kcal% protein for conventionalized (CVZ) mice and 18 kcal% protein for germ-free (GF) mice. Panel A (left) displays essential amino acids, while panel B (right) shows non-essential amino acids. For both panels, the x-axis represents the mean δ^13^C value of the Open Standard Diet proteins (n = 2) and the y-axis represents the mean δ^13^C value of CVZ (n = 5) or GF (n = 6) organs. The p-values indicate Pillai’s Trace from a one-way multivariate analysis of variance. There are no treatment effects except for the kidney non-essential amino acids at p < 0.05.

We plotted the δ^13^C-AA values of the sampled organs from both the GF and CVZ mice against the Open Standard diet to visualize tissue specific offsets relative to the diets and between the two treatments (Figs. 2A & B). We found no significant differences in organ-specific δ^13^C-EAA values between the two treatments (Pillai’s Trace, P > 0.05, Fig. 2A). However, we observed significant differences in δ^13^C-EAA values among the four organs (F_(9, 266)_ = 154.1, P < 0.0001), which held true for the six measured EAAs across organs in both dietary groups (F_(5, 270)_ = 118, P < 0.0001), with a significant interaction between dietary groups and amino acids (F_(45, 230)_ = 12.45, P < 0.0001). The δ^13^C-EAA values were highest in the liver, followed by the kidney, muscle, and brain (Table 1**A**). Among the EAAs, threonine (Thr) was the most enriched, while lysine (Lys) was the least (Table 1**B**). The similar δ^13^C-EAA values between GF and CVZ organs suggest limited gut microbial EAA supplementation in the CVZ mice. We found no treatment effects for non-essential amino acids except for the kidney at P < 0.05 (Fig. 2B).

**Figure 3.**
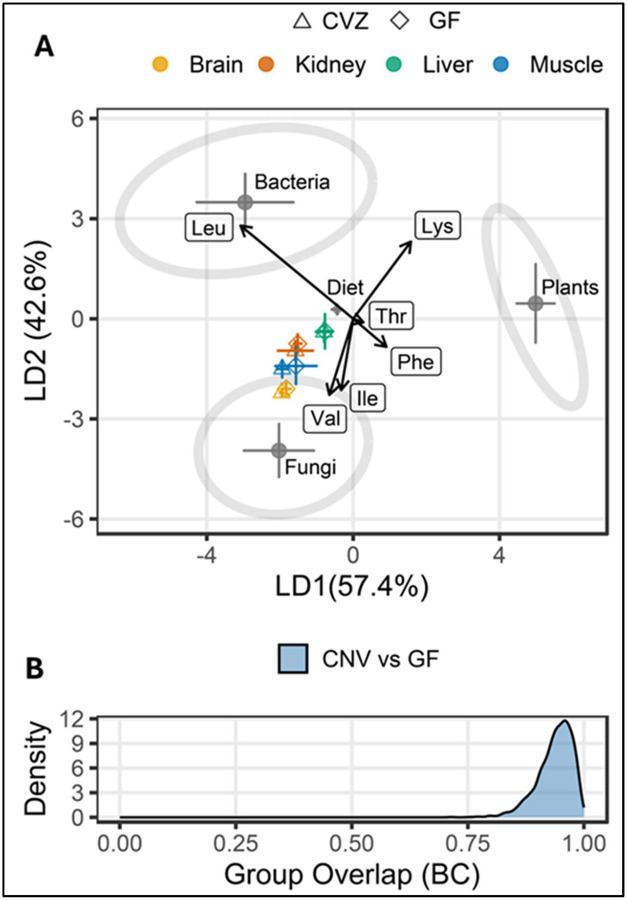
A) Linear discriminant function analysis (LDA) plot based on δ^13^C_EAA_ from GF-fed (N=5) and CVZ (N=5) and three classifier groups [fungi (N=9), bacteria (N=12) and plants (N=11)]. The shaded ellipses signify the 95% confidence limits for each classifier group. (B) Bhattacharyya coefficients (BC) quantifying group pair overlaps with 0 = no overlap and 1 = identical distributions. In this case, the median BC overlap between the two groups is 0.938, indicative of similar probabilistic distributions. Essential amino acids were Ile, isoleucine; Leu, leucine; Lys, lysine; Phe, phenylalanine; Thr, threonine; Val, valine.

Within the δ^13^C-EAA fingerprinting framework, the probabilistic distributions yielded a median Bhattacharyya coefficient (BC) value of 0.938, close to 1, indicating nearly complete overlap between GF and CVZ organs. This result shows that the predictive ordinations relative to the training data were almost identical for GF and CVZ across all organs (Figs. 3A and 3B), further supporting the absence of detectable microbial EAA contributions. See Table S1 and Table S2 for δ^13^C-EAA data generated in the study.

## Gut microbiome composition

Standard read quality-processing, ASV determination, and curation (removal of ASV assigned mitochondrion, chloroplast, and unassigned at the domain level) yielded 282 ASVs distributed across 10 samples. Rarefaction at 1,400 reads per sample resulted in a final ASV tally 171, with rarefaction curves indicating sufficient coverage across samples (Fig. S1). Initial cursory analyses indicated the dominance of contaminating Streptococcaceae (presumably from the diet) in GF-mice samples (unpublished communications from the Nebraska Gnotobiotic Mouse Program, NGMP). Subsequent removal of Streptococcaceae ASVs resulted in unacceptably low counts for the remaining ASVs in GF-mice samples, confirming the germ-free status of the GF mice at the end of the 20-day experimental period relative to CVZ mice. Thus, GF-mice samples were removed, and downstream analyses were performed for only individual CVZ mice.

**Figure 4.**
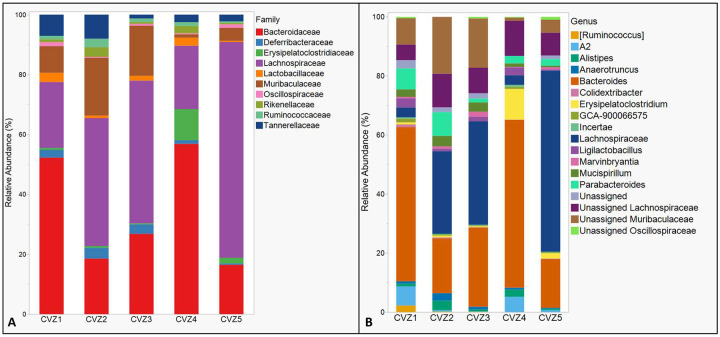
The relative abundances of the most abundant bacterial taxa in the fecal microbiomes of conventionalized (CVZ) mice at the end of the study at the (A) family level and (B) genus level.

Microbial richness (Chao1; 86.6 ± 4.88, and observed species; 75.8 ± 3.45, mean ± S.E) and evenness (Shannon’s index; 3.86 ± 0.20) estimates did not vary significantly among CVZ mice (P= 0.41 for all estimates). Lachnospiraceae (Phylum Firmicutes; abundant genus, Unassigned Lachnospiraceae) (32.92 %), Muribaculaceae (Phylum Bacteroidetes; genus Unassigned Muribaculaceae) (34.14 %), Bacteroidaceae (Phylum Bacteroidetes; genus Bacteroides) (10.18 %), Erysipelatoclostridiaceae (Phylum Firmicutes; genus Erysipelatoclostridium) (8.37%), and Rikenellaceae (Phylum Bacteroidetes; genus Alistipes) (4.67 %) were identified as dominant taxa in CVZ microbiomes (Figs. 4A & B). See Table S3 for the abundances of the topmost abundant 30 bacterial taxa in the fecal microbiome of CVZ mice at the end of the study.

## Discussion

Our study directly assesses gut microbial EAA provisioning in mice with reconstituted gut microbiomes following FMT by comparing the δ^13^C-EAA offsets and source diagnostic δ^13^CEAA patterns between GF and CVZ mice. Despite the determination of a typical reconstituted CVZ mice gut microbiome dominated by Firmicutes and Bacteroidetes, consistent with healthy mouse gut ecosystems, no evidence of significant microbial EAA provisioning after a 20-day feeding period was detected, failing to support our initial hypothesis. Furthermore, our δ^13^CEAA fingerprinting analysis revealed nearly complete overlap between GF and CVZ organs in δ^13^C pattern space, indicating that the predictive ordinations relative to the training data were almost identical for both groups across all organs. This findings contrasts with previous studies reporting substantial microbial contributions to host EAA pools in mice and other mammals with natural gut microbiomes^11,25^. The discrepancy between our findings and previous studies may be attributed to several key methodological differences.

First, our use of germ-free controls allowed for direct assessment of microbial contributions by isolating the microbiome as the sole variable. By comparing GF mice (lacking gut microbiota) with CVZ mice (containing reconstituted gut microbiomes), any differences in δ^13^C -EAA values could be directly attributed to microbial provisioning, eliminating confounding factors present when using mice with established microbiomes only. Our CVZ mice exhibited a typical gut microbiome composition dominated by Firmicutes and Bacteroidetes with Lachnospiraceae and Muribaculaceae as dominant families, consistent with healthy gut microbial ecosystems in omnivorous mammals fed low-protein, high-carbohydrate diets^33,34,48^. The diversity and complexity of dietary proteins can influence gut microbial composition and function, potentially affecting their capacity for EAA synthesis and provisioning. Interestingly, the prevalence of Lachnospiraceae, known for producing short-chain fatty acids (SCFAs) and associated with health benefits, did not translate to detectable EAA provisioning in our study. This observation aligns with a recent study suggesting that Lachnospiraceae may be more oriented towards carbohydrate metabolism and SCFA production rather than de novo amino acid synthesis^49^. Similarly, Muribaculaceae, which showed significant presence in our CVZ mice, have been reported to upregulate genes for carbohydrate metabolism rather than amino acid biosynthesis^50^.

Second, our methodological approach using a single protein source (lactic acid casein) with varying protein content (10% vs 18%) differs from other studies that employed diets with multiple protein sources and a wider range of protein content (9–40%)^25^. By using a single, well-defined protein source, our approach minimizes potential discrepancies between consumed and assimilated dietary proteins and reduces biases related to dietary memory effects^51,52^. Our findings with reconstituted gut microbiomes align with traditional understanding of mammalian digestive physiology, where protein digestion and amino acid absorption occur primarily in the small intestine, with limited absorption capacity in the colon where most gut microbes reside^11^. In contrast, another study reported substantial microbial EAA contributions to mouse muscle (between 15–40%) even when mice consumed high-protein diets (40%) that exceeded requirements for optimal growth^25^. However, in contrast with the current study, they used mice with naturally established wild microbiomes and diets containing C3-dominated protein sources and C4-dominated carbohydrate sources, creating different isotopic conditions compared to our controlled single-protein experimental design. The diversity and complexity of dietary proteins can influence gut microbial composition and function^53^, potentially affecting their capacity for EAA synthesis and provisioning. Our results suggest that the functional capacity for gut microbial EAA provisioning may be context-dependent, influenced by factors such as microbiome establishment history (native versus reconstituted) and dietary composition.

Finally, our focus on reconstituted rather than naturally established microbiomes may be significant, as the 30-day period following conventionalization prior to the feeding study may not have been sufficient for complete restoration of complex metabolic functions such as EAA biosynthesis and supplementation to host^26,32,54^. Previous studies have shown that while taxonomic composition can be quickly established following microbiota transplantation, functional restoration may follow different temporal dynamics, with some metabolic pathways requiring more extended periods to reach full capacity^26,31,32^. Our findings underscore the need for further research into the functional capacity of reconstituted gut microbiomes. Future studies should consider longer adaptation periods following FMT prior to diet switching studies, various protein restriction levels, and complementary approaches such as metatranscriptomics to assess microbial EAA biosynthetic pathway expressions. Additionally, comparative studies including both reconstituted and naturally established microbiomes would provide valuable insights into the functional equivalence of these systems and the potential ecological factors that enable or constrain microbial EAA provisioning in mammalian hosts. However, it is important to note that the methodology used in this study comparing δ^13^C offsets between GF and CVZ mice cannot be directly implemented with specimens having naturally established microbiomes since establishing a true germ-free control group is not feasible^55,56^. Comparative studies with positive controls would instead need to rely on alternative approaches such as isotope tracer additions (e.g., ^15^N and ^14^C labels used by Torrallardona et al. in pigs^11^) or δ^13^C-EAA fingerprinting with appropriate microbial end members to quantify potential microbial EAA contributions to host^57^. Recent advances in stable isotope resolved metabolomics (SIRM) using ^13^C-labeled dietary fibers like inulin could also provide valuable insights by tracking the dynamic flow of microbial metabolites, including amino acids, to host tissues across multiple organs^5^.

Overall, the absence of detectable microbial EAA provisioning in our CVZ mice raises important questions about the functional capacity of reconstituted gut microbiomes, the potential impacts of microbiome perturbations on host nutrition, and suitability of techniques to assess microbial functions. While our study focused specifically on EAA provisioning, the findings have broader implications for understanding microbiome functionality following gut microbiome reconstitution. The gut microbiome can be significantly altered by various factors, including diet, antibiotics, and environmental stressors, with potentially far-reaching consequences for host metabolism and nutrient acquisition^58,59^. Previous research has demonstrated that diet-induced extinctions in the gut microbiota can persist across multiple generations, potentially leading to permanent loss of specific microbial functions^60,61^. In the context of our study, this suggests that the lack of detectable EAA provisioning might reflect incomplete functional restoration in the conventionalized microbiome rather than an inherent inability of gut microbes to contribute EAAs to their hosts^61^. For example, studies with gnotobiotic mice have shown that colonization with gut microbiota from undernourished children can impair growth and metabolism, highlighting the critical role of the microbiome in nutrient utilization^62^. Our findings underscore the need for a more nuanced understanding of microbiome reconstitution, particularly regarding the restoration of complex metabolic functions that may require specific ecological conditions, microbial community structures, or longer adaptation periods than were provided in our experimental design.

## Conclusion

Despite being kept on a low-protein diet for 20 days, our murine model revealed no evidence of gut microbial EAA provisioning in conventionalized mice. This unexpected finding raises important questions about the functional capacity of reconstituted gut microbiomes and the potential impacts of microbiome perturbations on host nutrition. The lack of detectable EAA provisioning across multiple organs with varying turnover rates suggests that reconstituted microbiomes may not fully recapitulate the nutritional functions of native microbial communities. This sets the stage for more comprehensive studies to elucidate the complexities of gut microbial functions, the microbial dimensions of the nutritional ecology of omnivorous mammals, and our understanding of host-microbe interactions and the development of microbiome-focused therapies.

## Supplementary Material

1

## Figures and Tables

**Table 1.** Ingredients and macromolecular composition of purified diets fed to GF and CVZ mice.

Macronutrients	GF diet		CVZ diet	
Ingredients	g	kcal%	g	kcal%
**Protein (total)**	**16.5**	**18**	**9.2**	**10**
Casein	16.3		9.1	
L-Cystine	0.2		0.1	
**Carbohydrate (total)**	**61.7**	**66**	**69.9**	**74**
Corn Starch	36.2		42.8	
Maltodextrin	10.4		12.0	
Dextrose	14.2		14.4	
Sucrose	0.9		0.8	
**Fat (total)**	**6.6**	**16**	**6.7**	**16**
Soybean Oil	6.6		6.7	
**Fiber (total)**	**9.3**		**9.4**	**0**
Cellulose	6.98		7.05	
Inulin	2.32		2.35	

**Table 2.** Diet Normalized δ^13^C_EAA_ values (‰) in A) both the germ-free (GF) and conventionalized (CVZ) groups across the five organ types and in B) all six EAAs across organs. Significantly different means are represented by different letters.

A. Sampled Organs	Diet Normalized δ^13^C_EAA_ (%)
GF Muscle	4.2 (a)
GF Brain	3.9 (b)
CVZ Brain	3.8 (bc)
CVZ Muscle	3.6 (c)
GF Kidney	2.2 (d)
GF Liver	2.2 (d)
CVZ Kidney	2.05 (de)
CVZ Liver	1.92 (e)
CVZ Diet	0 (f)
B. Essential Amino acids	Diet Normalized δ^13^C_EAA_ (%)
Threonine	4.6 (a)
Isoleucine	3.6 (b)
Valine	3.3 (bc)
Leucine	3.2 (c)
Phenylalanine	2.6 (d)
Lysine	0.9 (e)

## Data Availability

The authors confirm that the data supporting the findings of this study are available within the article and its supplementary materials.
