# Supplementary material for: Assessing gut microbial provisioning of essential amino acids to host in a murine model with reconstituted gut microbiomes
Source: Res Sq. 2025 Mar 24:rs.3.rs-6255159. Preprint. [Version 1] doi: 10.21203/rs.3.rs-6255159/v1 (PMC11975013; doi:10.21203/rs.3.rs-6255159/v1)

Supplementary Tables

Table S1: Ingredients and macromolecular composition of pre-experimental diet, LabDiet® JL Rat and Mouse/Auto 6F (5K67).

**Macronutrients**	**Pre-experiment diet**	
Ingredients	**%**	**kcal%**
**Crude Protein (total)[1]**	**19.3**	**22.2**
**Carbohydrate (total)[2]**	**NA**	**61.7**
Starch	38.9	
Fiber (Crude)	4.3	
Neutral Detergent Fiber	15.1	
Acid Detergent Fiber	5.2	
Sucrose	0.6	
**Fat (total)[3]**	**6.2**	**16.0**

1) Ground wheat, ground corn, wheat middlings, ground oats, fish meal, dehulled soybean meal, dehydrated alfalfa meal, brewers dried yeast. 2) Ground wheat, ground corn, wheat middlings, ground oats, fish meal, dehulled soybean meal, corn gluten meal, dehydrated alfalfa meal. 3) Soybean oil, fish meal

Table S2: Mean δ^{13}C-AA values (n = 2) across six EAA obtained for the germ-free (GF) and conventionalized (CVZ) mice used in the study. The δ -notation is defined as [(R sample EAA /R standard EAA)-1] × 1,000 (per mill, ‰), where R is the ratio of heavy (^{13}C) to light (^{12}C) isotopes in a particular EAA of the sample R sample EAA and the standard R standard EAA [71].

Sample ID	Treatment	Ala	Asx	Glx	Gly	Tyr	Ile	Leu	Lys	Phe	Thr	Val
BCV1	Brain_CVZ	-12.6	-7.0	-5.4	-12.4	-29.3	-24.5	-29.3	-22.0	-29.4	-10.5	-26.0
BCV2	Brain_CVZ	-12.7	-7.0	-6.0	-11.1	-28.7	-24.3	-29.1	-21.7	-29.4	-10.3	-26.0
BCV3	Brain_CVZ	-12.7	-6.7	-5.7	-12.8	-28.3	-24.2	-29.0	-21.7	-28.8	-10.1	-25.6
BCV4	Brain_CVZ	-12.7	-8.5	-6.2	-12.9	-28.9	-24.3	-29.2	-21.6	-29.4	-10.8	-25.6
BCV5	Brain_CVZ	-12.2	-7.2	-6.1	-12.5	-28.8	-24.2	-29.2	-22.0	-29.4	-10.0	-25.7
BGF1	Brain_GF	-12.5	-7.4	-5.9	-11.9	-28.6	-24.4	-29.1	-21.6	-29.3	-10.0	-25.6
BGF2	Brain_GF	-12.7	-6.7	-5.2	-12.2	-28.9	-24.5	-29.3	-21.8	-29.4	-10.1	-25.6
BGF3	Brain_GF	-12.3	-6.8	-5.4	-12.2	-28.5	-24.0	-29.1	-21.0	-29.2	-9.4	-25.3
BGF4	Brain_GF	-12.5	-7.5	-5.9	-11.5	-28.0	-24.1	-28.6	-21.3	-28.9	-10.1	-25.8
BGF5	Brain_GF	-11.5	-6.4	-5.6	-11.8	-28.5	-24.1	-29.1	-21.4	-29.1	-10.0	-25.7
BGF6	Brain_GF	-12.3	-6.6	-5.2	-12.3	-28.5	-24.1	-29.0	-21.3	-29.0	-10.0	-25.4
KCV1	Kidney_CVZ	-12.1	-12.6	-9.3	-14.0	-30.2	-27.4	-31.1	-22.3	-30.6	-14.1	-28.0
KCV2	Kidney_CVZ	-12.4	-13.0	-10.0	-14.5	-30.3	-27.1	-30.7	-22.7	-31.0	-14.2	-27.8
KCV3	Kidney_CVZ	-9.3	-10.7	-9.6	-8.5	-27.6	-24.9	-29.7	-20.3	-29.4	-11.3	-26.1
KCV4	Kidney_CVZ	-14.4	-13.9	-10.9	-15.7	-30.9	-27.0	-31.0	-23.5	-31.4	-14.6	-28.0
KCV5	Kidney_CVZ	-12.7	-13.2	-10.4	-13.3	-30.0	-26.7	-30.5	-21.9	-30.6	-13.7	-27.6
KGF1	Kidney_GF	-12.2	-12.8	-9.9	-14.3	-29.3	-26.4	-30.4	-22.0	-30.1	-13.8	-27.4
KGF2	Kidney_GF	-12.4	-13.4	-10.3	-13.7	-29.7	-27.3	-30.5	-22.0	-30.5	-14.2	-27.9
KGF3	Kidney_GF	-14.0	-12.9	-10.0	-12.4	-29.2	-26.4	-30.2	-21.5	-30.3	-12.9	-26.9
KGF4	Kidney_GF	-14.2	-13.1	-10.3	-12.5	-28.9	-26.2	-30.1	-21.6	-30.1	-12.8	-26.8

KGF5	Kidney_GF	-13.9	-12.7	-10.2	-11.9	-29.2	-26.5	-30.4	-22.0	-30.3	-13.9	-27.7
KGF6	Kidney_GF	-12.7	-12.7	-10.1	-11.7	-29.1	-26.2	-30.2	-21.1	-30.2	-12.8	-27.0
LCV1	Liver_CVZ	-12.1	-12.2	-10.4	-12.7	-30.1	-27.6	-31.7	-22.9	-31.3	-12.1	-28.5
LCV2	Liver_CVZ	-12.2	-11.3	-9.7	-13.0	-29.0	-27.5	-31.4	-21.4	-30.6	-12.8	-28.2
LCV3	Liver_CVZ	-13.3	-11.3	-10.6	-11.4	-29.0	-27.5	-31.2	-21.4	-30.8	-11.0	-27.9
LCV4	Liver_CVZ	-12.3	-12.1	-9.8	-12.7	-29.1	-27.2	-31.0	-22.0	-31.0	-11.9	-28.7
LCV5	Liver_CVZ	-13.0	-12.3	-11.3	-11.4	-28.9	-27.5	-31.2	-22.1	-30.9	-11.4	-28.4
LGF1	Liver_GF	-12.1	-11.9	-10.4	-11.9	-28.5	-27.4	-31.1	-21.2	-30.6	-11.3	-27.9
LGF2	Liver_GF	-13.1	-11.8	-10.4	-12.0	-29.3	-27.4	-31.2	-21.7	-30.4	-11.4	-28.0
LGF3	Liver_GF	-11.5	-12.3	-10.5	-10.4	-28.5	-27.4	-30.6	-21.5	-30.4	-10.7	-29.0
LGF4	Liver_GF	-12.9	-11.5	-10.6	-11.9	-29.1	-27.2	-30.8	-21.9	-30.4	-11.3	-28.1
LGF5	Liver_GF	-13.2	-11.7	-10.8	-12.2	-29.4	-27.3	-31.1	-22.1	-30.8	-11.0	-27.7
LGF6	Liver_GF	-13.4	-12.4	-11.3	-12.4	-29.5	-27.2	-31.0	-22.2	-30.5	-11.3	-27.7
MCV1	Muscle_CVZ	-14.3	-15.4	-13.1	-13.3	-27.9	-24.0	-29.1	-21.4	-29.3	-12.8	-27.0
MCV2	Muscle_CVZ	-15.0	-15.2	-13.2	-13.4	-27.8	-23.6	-28.8	-21.3	-29.0	-13.1	-26.4
MCV3	Muscle_CVZ	-13.6	-15.9	-13.9	-13.0	-27.0	-23.1	-28.8	-20.9	-28.9	-12.1	-27.0
MCV4	Muscle_CVZ	-15.8	-16.6	-14.1	-14.5	-27.7	-23.9	-28.6	-21.3	-27.3	-13.3	-26.2
MCV5	Muscle_CVZ	-15.2	-15.8	-13.7	-13.1	-27.5	-23.7	-28.5	-20.5	-28.2	-12.5	-26.3
MGF1	Muscle_GF	-13.4	-15.5	-13.9	-10.8	-27.4	-23.6	-28.3	-19.3	-28.0	-12.1	-26.3
MGF2	Muscle_GF	-15.5	-15.1	-13.1	-13.1	-26.9	-23.5	-28.4	-20.4	-27.4	-12.0	-25.8
MGF3	Muscle_GF	-14.6	-15.9	-14.1	-12.6	-28.2	-23.5	-28.2	-20.5	-26.1	-12.3	-26.1
MGF4	Muscle_GF	-14.9	-16.1	-14.5	-13.3	-27.9	-23.6	-28.2	-21.4	-28.3	-12.6	-25.7
MGF5	Muscle_GF	-15.4	-15.4	-13.7	-12.2	-27.6	-23.4	-28.2	-18.9	-26.6	-11.5	-25.6
MGF6	Muscle_GF	-14.9	-15.8	-13.8	-13.5	-27.4	-23.6	-28.4	-21.0	-27.5	-12.4	-25.7
IVC	CVZ Diet	-24.2	-22.1	-20.5	-18.9	-30.4	-29.0	-33.0	-22.6	-32.2	-16.4	-30.1
ISO	GF_Diet	-23.8	-21.8	-20.3	-18.2	-30.4	-29.0	-33.0	-22.2	-32.1	-16.5	-30.1

Table S3. The abundances of the 30 differentially abundant bacterial taxa in the fecal microbiome of CVZ mice at the end of the study, distributed across 10 bacterial family-level and eighteen genera.

**Family**	**Genus**	**CVZ1**	**CVZ3**	**CVZ2**	**CVZ5**	**CVZ4**
Bacteroidaceae	*Bacteroides*	641	246	209	201	682
Muribaculaceae	*Unassigned Muribaculaceae*	109	153	220	53	14
Tannerellaceae	*Parabacteroides*	91	12	94	28	31
Lachnospiraceae	*A2*	83	1	5	8	64
Lachnospiraceae	*Unassigned*	36	17	19	15	0
Lachnospiraceae	*Unassigned Lachnospiraceae*	35	62	96	67	108
Deferribacteraceae	*Mucispirillum*	33	30	42	6	15
Bacteroidaceae	*Bacteroides*	30	6	8	2	19
Lachnospiraceae	*[Ruminococcus]*	29	1	2	0	0
Lachnospiraceae	*Lachnospiraceae*	23	215	199	492	18
Lactobacillaceae	*Ligilactobacillus*	22	8	4	3	20
Rikenellaceae	*Alistipes*	12	6	38	6	30
Lachnospiraceae	*Unassigned Lachnospiraceae*	12	7	20	8	29
Lachnospiraceae	*Lachnospiraceae*	12	4	4	11	12
Lachnospiraceae	*GCA-900066575*	12	3	3	2	6
Lactobacillaceae	*Ligilactobacillus*	12	4	3	1	8
Lachnospiraceae	*Unassigned Lachnospiraceae*	11	6	6	12	8

Oscillospiraceae	*Colidextribacter*	11	1	4	2	1
Ruminococcaceae	*Anaerotruncus*	10	9	30	4	8
Lachnospiraceae	*Unassigned Lachnospiraceae*	10	6	11	8	1
Erysipelatoclostridiaceae	*Erysipelatoclostridium*	9	3	7	23	128
Lachnospiraceae	*Lachnospiraceae*	8	110	124	253	10
Lachnospiraceae	*Marvinbryantia*	6	16	9	9	2
Lactobacillaceae	*Ligilactobacillus*	6	3	3	1	5
Oscillospiraceae	*Unassigned Oscillospiraceae*	6	5	0	12	3
Ruminococcaceae	*Incertae*	5	2	3	2	8
Lachnospiraceae	*GCA-900066575*	5	0	1	2	3
Muribaculaceae	*Unassigned Muribaculaceae*	5	4	5	1	0

Figure S1. Rarefaction plots (1400 reads per sample) showing coverage depth and higher microbial richness estimates for conventionalized, CVZ mice relative to germ-free, GF mice.

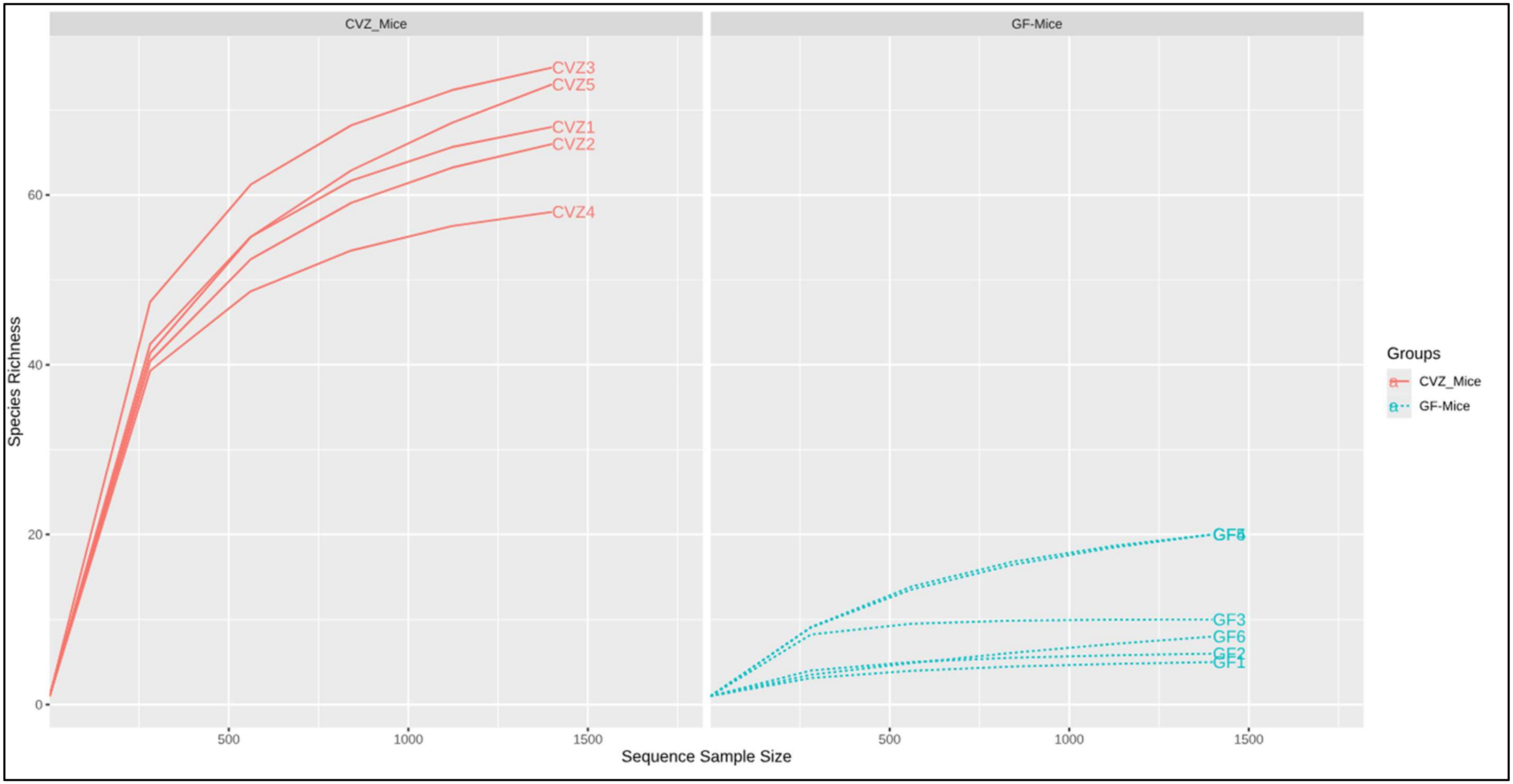

Supplement: 1 [file NIHPPrs6255159V1-supplement-1.pdf]
